# ML-based tooth shade assessment to prevent metamerism in different clinic lights

**DOI:** 10.1007/s10103-025-04297-y

**Published:** 2025-01-24

**Authors:** Abdullah Ammar Karcioglu, Esra Efitli, Emrah Simsek, Alper Ozdogan, Furkan Karatas, Tuba Senocak

**Affiliations:** 1 Present Address: Atatürk University, 25240 Erzurum, Turkey; 2 Erzurum Technical University, 25240 Erzurum, Turkey; 3 Iğdır Üniversitesi, 76000 Iğdır, Turkey; 4 Erzincan University, 24002 Erzincan, Turkey

**Keywords:** Image Processing, Machine learning, Metamerism, Vita 3D Master, Prosthodontics

## Abstract

The aesthetic understanding has found its place in dental clinics and prosthetic dental treatment. Determining the appropriate prosthetic tooth color between the clinician, patient and technician is a difficult process due to metamerism. Metamerism, known as the different perception of the color of an object under different light sources, is caused by the lighting differences between the laboratory and the dental clinic. The traditional trial-error color determination method, coupled with the high cost of instrumental color value determination, has prompted the need for alternative technologies. The integration of AI technologies into dental practices aims to minimize errors in tooth shade assessment, reduce equipment usage, eliminate the impact of clinic lighting on color detection, and decrease costs for patients, dentists, and laboratories. In this study, a machine learning (ML) based approach that can correctly detect tooth shade even under different clinical lights has been developed. A dataset consisting of 580 dental images taken under four different clinical lights and with five repetitions was created using the Vita color shade guide. Experimental studies were performed using the HSV color space, 6 different ML algorithms and color histograms. As a result, 97.93% accuracy rate was achieved by using cross-validation (cv = 5) in the classification of 29 color values ​​independent of clinical lights. It has been shown that the tooth colors can be determined with high accuracy using ML algorithms and metamerism can be prevented.

## Introduction

Visual appearance (aesthetics) provides perceptual comfort to the person and is also used in prosthetic dental treatment. The exact detection of tooth shades and their transfer to the laboratory are the most important esthetic parameters [1]. Color in dental prosthetics is the quality of an object or substance in relation to the reflected or transmitted light [2] and color, according to the definition of physics, is light [3]. Tooth shade is detected by the paths of light within the tooth, which are determined by light scattering, and the absorption of light along these paths [4]. Clinically, color detection is a subjective process that is influenced by factors such as light source, object and observer [5]. Determining the appropriate prosthetic tooth shade is a difficult process due to metamerism. Metamerism, which means the color of an object is perceived differently under different light sources, is a phenomenon that can result from different lighting in the laboratory and dental clinic [6]. There are other factors that make and influence color selection in clinics subjective: the patient, the environment, the nature of the tooth, the lighting conditions [7, 8], the background [9], stains that may appear on the surface of the tooth and dentist’s decision [7, 8]. Since the color of the natural tooth and the color of the prosthetic tooth are different shades of white, they will be perceived as the same after a while. This perception, which is caused by eye fatigue and the external conditions, leads to an incorrect choice of shade and thus to the desired esthetic perception being exceeded.

**Fig. 1:**
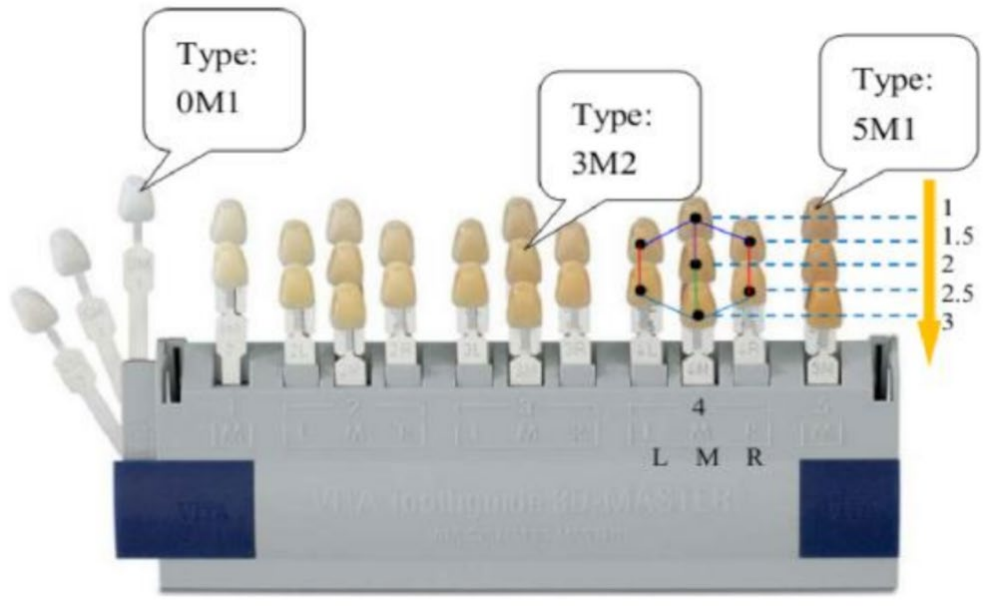
The Vitapan 3D master shade guide [14]

The methods for identifying tooth shade in restorative dental treatment can be divided into two areas: visual and instrumental methods. Visual method (with shade guide) is the most commonly used method for color matching in clinical practice [10]. Although there are various shade guides on the market, the most commonly known are Vita Classic and Vita 3D Master, as shown in Fig. 1. This visual process can lead to inconsistencies in color selection and treatment failure [11]. Some studies have shown that it is difficult to match the shade tabs to the teeth in the intraoral environment [12, 13].

Instrumental methods (Spectrophotometers, colorimeters) enable the transition from subjective to objective analysis of color and instrumental devices lack of standardization, are very expensive and have relatively low performance [15].

In this study, color matching of dental images was performed using ML algorithms. This technology-based method simplifies the recording and matching of colors in dental practices. The aim is to select tooth color independently of clinical lighting. In the literatüre, color matching which provides accurate results under different clinical lighting conditions, was not mentioned. In our study, dental color matching was designed to be compatible with different clinical light sources to prevent the color from changing depending on the light source. We can list our contributions to this study as follows:

- ML techniques were employed instead of traditional methods.
- Tooth colors were determined independently of the clinic’s lights.
- In dentistry has demonstrated the ability to prevent metamerism.
- Color selection errors caused by human factors are minimized.
- Different clinical lights are classified with an accuracy of 99.83%.
- Different clinical lighting conditions, 29 tooth shades are classified with 97.93% accuracy.

Section 2 mention the literature. Section 3 explaine the methods and materials. Section 4 presents the experimental results. Section 5 draws the conclusion.

## Literature review

Jarad et al. [16] compared the traditional method and the computer-assisted color matching method. Some studies have shown that the smartphone can be used as an instrument for color measurement in the dental clinic [13, 17]. In addition, in studies where shade guides were used, it was observed that multiple cameras were used due to the limited color values of the scales or excessive repetition of the same color tab was used to increase the amount of data [7, 14, 18, 19, 20]. In another study, the color value selected as a reference from the shade guide and the target tooth whose value is desired were taken together [17].

A summary of the fully technology-driven and artificial intelligence-based color matching studies is shown in Table 1. RGB, HSV and Lab are the color spaces used in the literature. The effect of color spaces on accuracy varies between studies in the literature. Support Vector Machines (SVM), Logistic Regression (LR), K-nearest neighbor (KNN), Random Forest (RF), Decision Trees (DT), Neural Network (NN) and Fuzzy Logic [8, 21] are artificial intelligence methods used in the literature. Vita classic and Vita 3D master are used image sources.

**Table 1:** Summary of studies on tooth shade recognition based on ML

Reference	Color Space	Image Source	Model	Data Size	Accuracy
[7]	Hsv-Lab-Rgb	Vitapan 3D Master	Knn	1300	70%
[8]	Ycbcr-Cielab	Vita 3D Master	Fuzzy	26	92.31%
[13]	Rgb-Hsv-Xyz-Lab	Vita 3D Master	Svm	1300	Group 1:86- 98%, Group 2: 97– 100%
[14]	Hıs	Vita 3D Master	Knn	29(vita)*20(repeat) + 3(class)*20	90%
[17]	-	Vita Classic	Rf, Svm, Xgboost	15.360	Rf: 97.1%
[18]	Rgb-Hsv-Lab	Vitapan Classical, image of patients	Knn, Nn, Dt	640	Knn: 97.5%, Nn: 76.8%, Dt: 85.63%
[19]	Rgb-Hsv-Lab	Vita Classic	Knn	Train: 576, Test: 64	Rgb: 97.8%, Hsv: 65%, Lab: 94.8%
[20]	Cielab	Vita 3D Master, image of patients	Svm, Lr, Rf, Knn	Train: (26*5 camera) Test: (26*3)	Svm: 96.9%, Lr: 77.2%, Rf: 89.3%, Knn: 96.1%
[22]	Hsv-Rgb	Vita, image of patients	Nn	62 image, 816 data	68%
**Our Study**	Hsv	Vita 3D Master	Rf, Knn, Linear SVM, Linear SVR, NonLinear SVM, NonLinear SVC	580 = 29 (class) * 5(repeat) * 4(clinic lights)	**Independent of clinic lights** 29 vita shade: **97.93%**4 clinic Light: **99.83%** 29 vita shade in white light:**100%** 29 vita shade in natural light: **99.31%** 29 vita shade in flash light: **96.55%** 29 vita shade in yellow light: **95.86%**

## Method and materials

In this study, HSV color space, color histograms and ML algorithms were used to determine the best model for color matching in dental images. The algorithms are implemented in the Python programming language.

### The dataset

The tooth shade is usually matched visually using a shade guide. Vita Classical and Vitapan 3D Master are the most known shade guides in dentistry. Vitapan 3D Master is similar to the Munsell value, which represents the three dimensions of color [23]. In this study, Vita 3D Master, which has more colors, was chosen as the image source.

This in vitro study used a smartphone camera to collect images. The images were captured using the iPhone 13 Pro Max in photo mode with 3x zoom. Before capturing each image, the focus was adjusted and fixed on the corresponding tooth to ensure accurate imaging. Each color tab on the Vita was captured against a gray background in four different clinical light conditions, with 5 replicates for each environment. These four different clinical light sources are: natural light source without flash support (natural), flash light under natural light source (flash), light in dental unit without flash support (white), yellow light source in dental units without flash support (yellow). The temperature of these lights was measured to range from 2700 K to 6500 K. A tripod setup equipped with an LED lighting system was used as the light source. The distance between the tripod and the samples was generally set to 20 cm, and the images were captured in a standardized manner. The light source was specified to have a total power of 84 watts, a light output of 11,300 lumens, a CRI value of 96, and 480 LEDs.

**Fig. 2:**
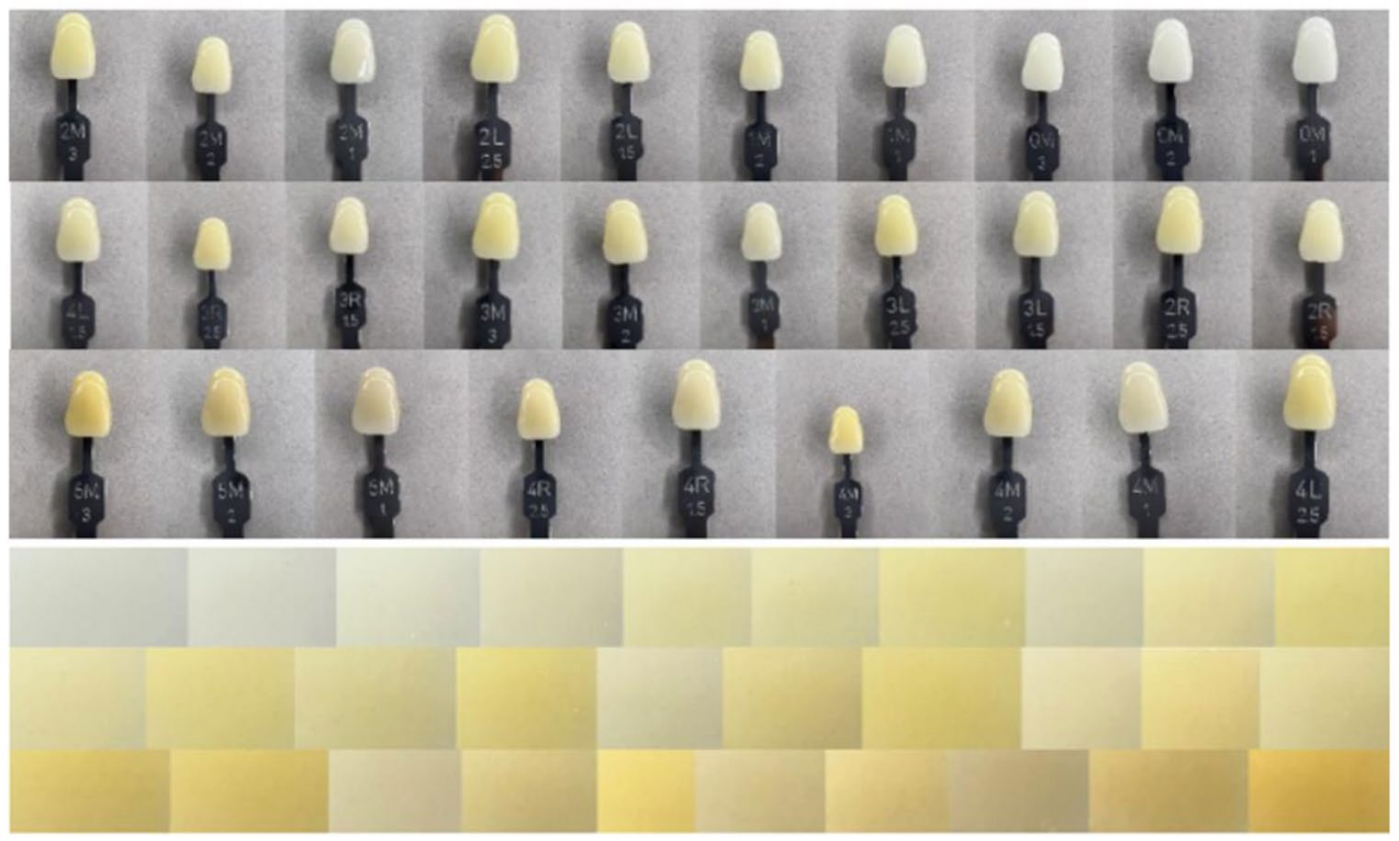
Vita 3D master and cropped images

Each image was cropped from the equatorial region where the tooth shade is most pronounced, regardless of size. A dataset of cropped images was created. Sample images of each tooth on the Vita and cropped images are shown in Fig. 2. Color histograms were used to images processing and represent the images numerically. These numerical values ​​are the input values ​​for the ML algorithms. The name of each image represents the color value of the images and the folder names represent the clinical light in which it was captured. These labels were included in the dataset after the images were processed.

### ML-based tooth shade assessment using color histograms

Color histograms show the range and frequency in which pixel values ​​of images are observed. In this classification process, bins (64,64,64) were used, which classify the image pixels with boxes of specified size. Accuracy results were obtained for six different classifications:

- 4 different clinical lighting conditions.
- 29 colors independent of light sources.
- In white light, 29 colors.
- In natural light, 29 colors.
- In yellow light, 29 colors.
- In flash light, 29 colors.

In this approach, HSV color space is used. Cross-validation was performed for classification and a k value was chosen as 5. The training/test split was 70/30. The data was normalised. ML algorithms were used.

**Fig. 3:**
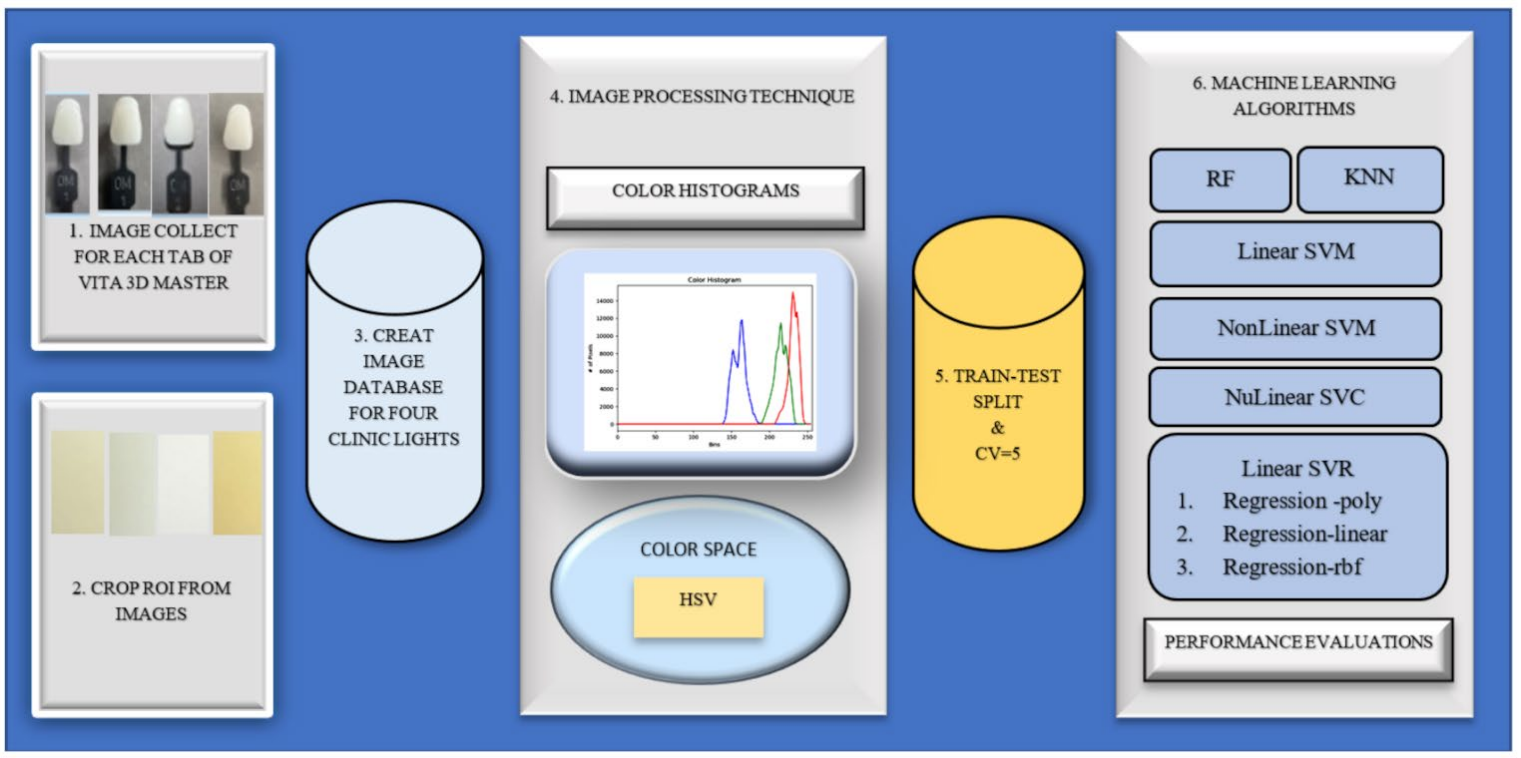
The general framework of this study

Support Vector Machine (Linear SVM) is an algorithm used to classify data in a linear fashion, determining class boundaries with maximum distance. Support Vector Regression (Linear SVR) is a regression algorithm used to find the optimal linear relationship between the target variable and independent variables in linear relationships. NonLinear SVM classifies non-linear data using kernel methods and can learn more complex boundaries. NonLinear Support Vector Classifier (NonLinear SVC) is the classification version of NonLinear SVM and uses kernel functions to classify non-linear data. K-Nearest Neighbor (KNN) classifies data by looking at the nearest neighbors of each data point to predict the class. Random Forest (RF) is a powerful ensemble learning algorithm where multiple decision trees are combined to make a collective prediction. The general framework of this study is shown in Fig. 3.

### Performance metrics

In this study, we used several performance metrics for evaluate the model. True Positive (TP) refers to correctly classified positive observations, True Negative (TN) refers to correctly classified negative observations. False positive (FP) refers to false classified positive observations and false negative (FN) refers to false classified negative observations. Accuracy is the ratio of correctly classified predictions to all predictions [24] and its formula is given in Eq. 1. Recall, precision, F1-score values are calculated for each class. Since 29 classes were used for color detection in this study, the results of these performance values reduce the readability in the tables. For this reason, the performance analysis of this study was conducted using the accuracy metric.

1 $$\:Accuracy=\frac{TP+TN}{TP+TN+FP+FN}$$

Other performance metrics used in classification problems are confusion matrix. Confusion matrices are obtained by comparing the predicted value in the test data with the actual value. Correct predictions are collected on the diagonal of the matrix. The size of the matrix is a square matrix with row and column size equal to the number of classes [25]. In cases where data is limited, test data may not include every class. In this case, as many matrices are formed as there are classes in the test data. Therefore, it is important that the dataset contains enough examples for each class.

## Experimental results

As shown in Table 2, high performance was obtained in color recognition regardless of the light in which it was taken. In the train/test dataset splitting, notable achievements were observed. Notably, the highest accuracy rate in classifying four different clinical lights reached 97.13% with Nonlinear SVC, whereas for the classification of 29 color values, the accuracy rate peaked at 92.53% with Nonlinear SVC. Impressively, in images captured under white light, the classification accuracy for 29 colors achieved perfection at 100% accuracy with Nonlinear SVC. Similarly, in NonLinear SVC, the classification accuracy rates of 29 colors in natural, flash and yellow light conditions remained as high as 97.73%.

**Table 2:** Classification results (bins: (64,64,64)

Split Rate	Classification Approaches		Algorithms							
			Linear SVM	Linear SVR (Reg- poly)	Linear SVR (Reg- linear)	Linear SVR (Reg-rbf)	NonLinear SVM	NonLinear SVC	KNN	RF
**Train: %70** **Test: %30**	Light Source Independent	4 Class Accuracy [Clinic Light Classification]	94.83%	87.91%	84.85%	87.53%	79.31%	**97.13%**	93.68%	90.23%
		Accuracy of 29 Class [Vita Color Tabs]	87.36%	69.55%	73.06%	87.13%	62.07%	**92.53%**	82.76%	78.16%
	Light Source Dependent	Accuracy of 29 Class [White]	95.45%	97.54%	97.59%	98.19%	86.36%	**100.00%**	88.64%	86.36%
		Accuracy of 29 Class [Natural]	97.73%	96.51%	96.97%	97.05%	65.91%	**97.73%**	88.64%	97.73%
		Accuracy of 29 Class [Flash]	97.73%	92.53%	95.32%	94.64%	72.73%	**97.73%**	86.36%	86.36%
		Accuracy of 29 Class [Yellow]	86.36%	53.88%	79.42%	84.50%	75.00%	**97.73%**	72.73%	86.36%
**CV:5**	Light Source Independent	4 Class Accuracy [Clinic Light Classification]	98.62%	95.07%	93.20%	94.94%	87.07%	**99.83%**	98.10%	95.86%
		Accuracy of 29 Class [Vita Color Tabs]	96.03%	81.23%	73.86%	89.08%	75.00%	**97.93%**	94.31%	89.66%
	Light Source Dependent	Accuracy of 29 Class [White]	99.31%	98.39%	97.76%	98.65%	99.31%	**100.00%**	**100.00%**	99.31%
		Accuracy of 29 Class [Natural]	**99.31%**	97.35%	97.92%	97.91%	**99.31%**	**99.31%**	**99.31%**	**99.31%**
		Accuracy of 29 Class [Flash]	95.86%	92.66%	90.06%	92.64%	94.48%	**96.55%**	95.17%	95.17%
		Accuracy of 29 Class [Yellow]	**96.55%**	61.25%	75.54%	81.73%	**96.55%**	95.86%	95.86%	93.10%

As shown in Table 2, the cross-validation results further corroborated the efficacy of the color histogram. This notable accuracy rates are particularly noteworthy given the complexity of distinguishing among 29 classes. Herein, the highest accuracy rate in classifying four different clinical lights soared to 99.83% with Nonlinear SVC, while for 29 color values, it peaked at 97.93% with Nonlinear SVC. Noteworthy is the perfect accuracy achieved in classifying 29 colors in images captured under white light, emphasizing the robustness of the approach under controlled lighting conditions. Similarly, in natural light scenarios, the classification accuracy for 29 colors remained impressively high at 99.31% with Nonlinear SVC. However, in instances of flash and yellow light, while maintaining competitive accuracy rates, slight variations were observed, with accuracy rates of 96.55% with Nonlinear SVC and Nonlinear SVM, respectively.

Generally, superior outcomes were achieved with the NuLinear SVC classification algorithm. Notably, the best results are highlighted in bold in Table 2. The results indicate that all approaches consistently deliver classification with a high level of accuracy. This suggests that the application of artificial intelligence has the potential to effectively mitigate metamerism-related issues.

**Fig. 4:**
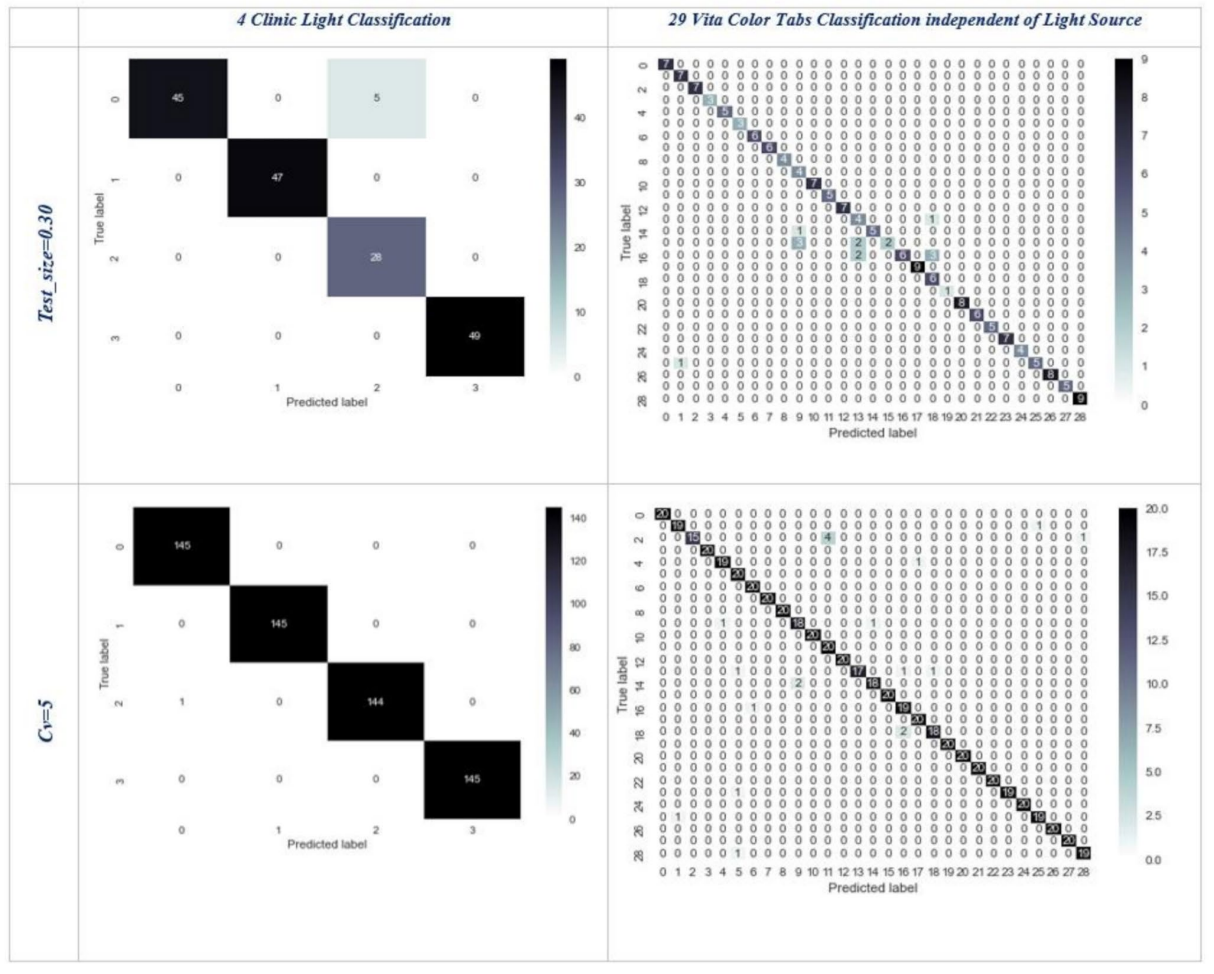
Confusion matrices of the best accuracy rates independent of the light source

**Fig. 5:**
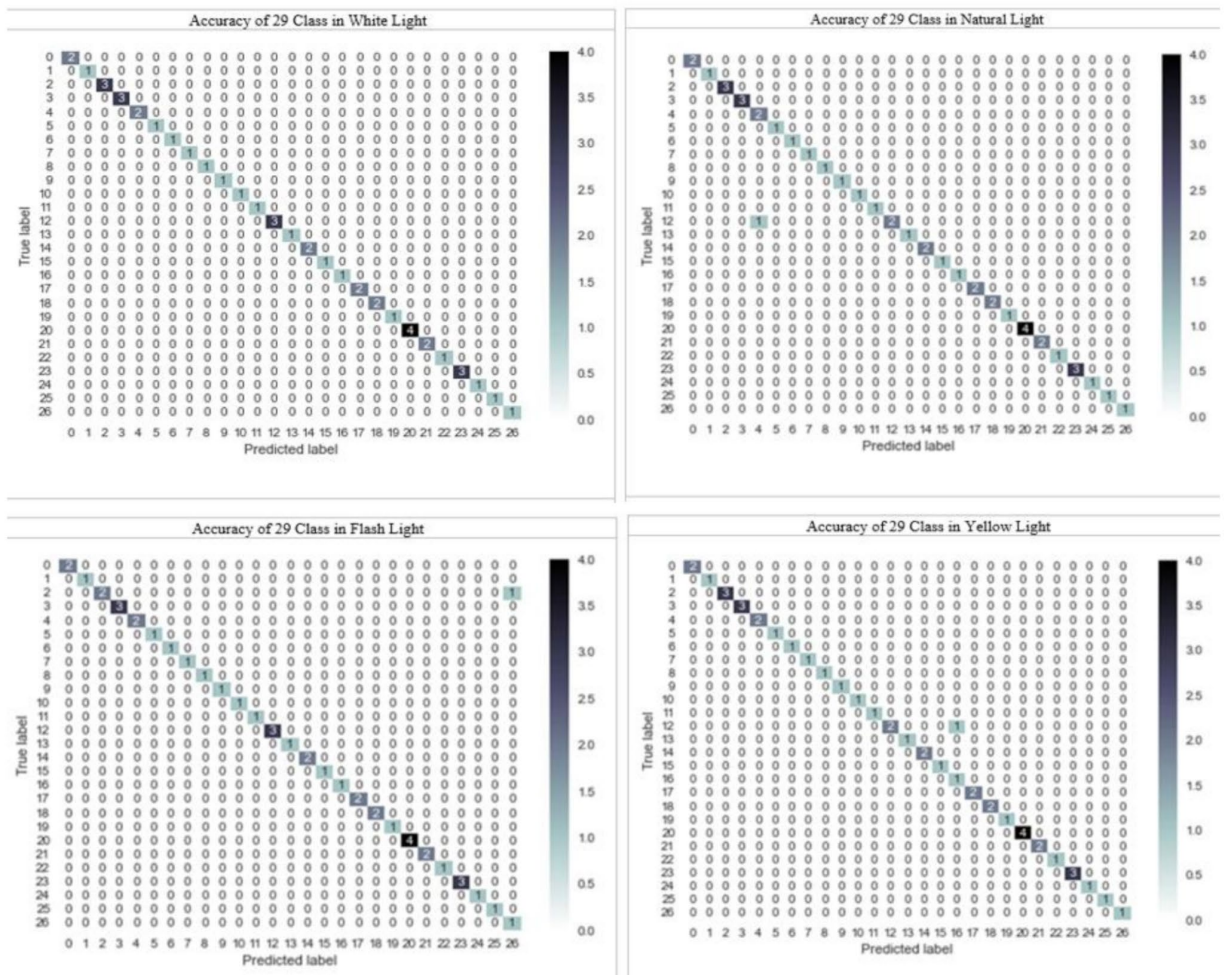
Confusion matrices of the best accuracy rates dependent on the light source

The confusion matrices corresponding to the highest accuracy values obtained are illustrated in Figs. 4 and 5. The confusion matrices for the classification of different clinical lights and the classification of 29 different colors independent of clinical lights are presented in Fig. 5. Notably, in the classification of clinical lights, only five test data points were inaccurately estimated. However, upon selecting cv = 5 for the same classification, the confusion matrix revealed just one incorrectly estimated value, demonstrating a near-perfect result. Similarly, enhanced outcomes were observed for cv = 5 in the classification of 29 colors.

Figure 5 illustrates the confusion matrix corresponding to the highest accuracy rates obtained across 29 different color classes of images categorized according to four different clinical lights. Each clinical light classification comprises 145 images. Due to the limited number of examples in each subset for cv = 5, the confusion matrices for these classifications are presented based on model training with a test size of 30%. Additionally, considering the possibility of the test data not encompassing all 29 classes due to data scarcity, the confusion matrices are depicted based on the number of classes present in the test data. As shown in Fig. 5, it was observed that all predictions were accurately made for images captured under white clinical light. Similarly, for images captured under natural, flash, and yellow light sources, only one data point was inaccurately estimated. These results demonstrate near-perfect matches between predicted and actual values.

## Conclusion

The traditional method used to determine the color of prosthetic teeth is subjective and instrumental measurement is costly. In this study, tooth color selection was made with ML, independent of clinical lighting.

The dataset consisting of 580 dental images was collected with 29 prosthetic tooth samples in the Vita 3D master shade guide, captured five times in four different clinic lights. Color histograms have been applied to detect dental color with ML algorithms.6 ML algorithms (Linear SVM, Linear SVR (Regression-poly, Regression-linear, Regression-Rbf), Nonlinear SVM, NonLinear SVC, KNN, RF) were used. NonLinear SVC algorithm achieved the best accuracy rate of 97.93% using the cross-validation method (k = 5).

The classification of clinical lights accuracy rate is 99.83%. This accuracy rate shows that clinical lights have high discrimination in the computer environment. In the classification of 29 colors in each of the clinic lights, a 100% accuracy rate was achieved with the white clinical light and an accuracy rate close to this value in other classifiers, showing that the use of white light in clinics is appropriate for tooth shade assessment.

Although shade guides are standardized, they are known to differ from natural teeth in color representation. This study has taken this limitation into account by utilizing the Vita 3D Master, which has the widest range of shades, and its applicability to clinical practice is not a problem. However, with the production of wider shade guides, the study may become even more comprehensive in the future.

In this study, it was shown that objective and low-cost determination of color value is possible. The ease of use, low cost compared to laboratory equipment, portability, and fast data collection capability of mobile devices increase the practical applicability of the method. However, calibration and user experience are important for accuracy. Compared to studies in the literature, the inclusion of different clinical lights did not reduce the accuracy rate and a better accuracy rate was obtained than most studies. In addition, in vivo studies are essential to demonstrate the applicability to routine tooth color determination and to confirm the clinical relevance. In future will focus on an in vivo study using real patient teeth.

## Limitations

The limitations of this study are as follows: The shade guides are made of layered ceramic materials. Although the ceramic color samples have been standardized, they may exhibit differences in color compared to natural teeth. Further in vivo studies could be conducted to validate the clinical significance.

## Data Availability

No datasets were generated or analysed during the current study.
